# Novel aspect of oxytocin neurons mediating parental behavior and aversive burying behavior under the control of melanin-concentrating hormone neurons

**DOI:** 10.3389/fnbeh.2024.1459957

**Published:** 2024-09-23

**Authors:** Tingbi Xiong, Lena Tsuchida, Ayumu Inutsuka, Tatsushi Onaka, Kazuo Yamada, Chitose Orikasa

**Affiliations:** ^1^Laboratory for Psychology and Behavioral Neuroscience, University of Tsukuba, Tsukuba, Japan; ^2^Department of Physiology, Jichi Medical University, Shimotsuke, Japan; ^3^Laboratory for Morphological and Biomolecular Imaging, Nippon Medical School, Tokyo, Japan

**Keywords:** oxytocin, melanin-concentrating hormone, parental behavior, burying, optogenetics

## Abstract

Parental behavior comprises a set of crucial actions essential for offspring survival. In this study, a double transgenic mouse model engineered to specifically express channelrhodopsin-2 (ChR2) in paraventricular hypothalamic nucleus (PVN)–oxytocin neurons and ablate lateral hypothalamic area (LHA)–melanin-concentrating hormone (MCH) neurons was used to determine the relationship between PVN–oxytocin neurons and LHA–MCH neurons associated with parental behavior. Optogenetic stimulation of ChR2-expressing PVN–oxytocin neurons induces typical parental behavior with intact LHA–MCH neurons. However, after the partial ablation of LHA–MCH neurons, even optogenetic stimulation of PVN–oxytocin neurons failed to induce parental behavior in virgin male mice, resulting in neglect rather than parental behavior. Furthermore, approximately half of the subjects exhibited burying behavior toward pups, suggesting that pups became aversive stimuli, and male mice actively performed burying behavior to avoid these aversive stimuli. This study emphasizes the novel aspect of oxytocin neurons that could result in neglect in the absence of LHA–MCH neurons regulation.

## 1 Introduction

Pregnancy and birth experiences alter the responsiveness of female rodents to pups. For instance, virgin rodents typically exhibit avoidance toward pups rather than displaying maternal behavior initially (Fleming and Rosenblatt, 1974). When pups are placed near a virgin female’s nest, it will actively avoid the pups and rebuild the nest elsewhere (Fleming and Luebke, 1981). However, those with birth experience (known as dams) display caregiving behavior compared with virgin females. For instance, dams quickly approach pups and engage in a typical set of maternal behaviors (Fleming and Rosenblatt, 1974). Interestingly, in the case of priming with pups’ exposure, virgin females exhibit parental behavior toward foster pups. For example, virgin females cohoused with a dam and her pups could display typical pup retrieval behavior, a phenomenon that can be reproduced by the central administration of oxytocin (Pedersen et al., 1982).

In contrast, virgin male mice exhibit varying responses to pups, typically demonstrating aggression, which often results in infanticide. Nevertheless, when they spend time with pregnant females after mating, they become less aggressive and display parental tendencies (Tachikawa et al., 2013). We have previously demonstrated that even virgin male mice can exhibit parental behavior when exposed to certain social environments (Orikasa et al., 2015), and the interaction between lateral hypothalamus area (LHA)–melanin-concentrating hormone (MCH) neurons and paraventricular hypothalamic nucleus (PVN)–oxytocin neurons is essential for regulating parental behavior in virgin male mice (Kato et al., 2021). MCH, a neuropeptide first isolated from the pituitary of teleost fish, is also found in the hypothalamus of rodents (Vaughan et al., 1989). This ubiquitous peptide has various functions within the central nervous system, such as homeostatic regulation, sleep, feeding behavior, motivation, learning, plasticity processes, emotion and reproduction (Concetti et al., 2024). It has been suggested that inputs from MCH neurons to PVN-oxytocin neurons are potentiated after mice become fathers (Inada et al., 2022).

Oxytocin is a neuropeptide produced in the hypothalamus with an inherent physiological function such as in parturition and lactation during nursing, and it also acts on social behaviors, including parental behavior in rodents. The literature reports valuable findings regarding the neural mechanisms underlying oxytocin regulation of maternal behavior (Marlin et al., 2015). Social stimuli can induce the secretion of oxytocin (Kato et al., 2021), which in turn, leads to the expression of maternal behavior. Moreover, optogenetic stimulation of PVN–oxytocin neurons can induce parental behavior in mice (Marlin et al., 2015).

It assumed that virgin males commit infanticide during their first encounter with pups; however, social stimuli elicit and change behavior characteristics such as parenting in virgin males (Orikasa et al., 2015). Neural circuits responsible for infanticide could easily shift to the mode of parental behavior. Animals with congenitally ablated MCH neurons under the control of the Tet-off system exhibited reduced parental behavior among females, and more aggressiveness toward pups among males (Kato et al., 2021). Therefore, in virgin males, it is possible to conduct a detailed analysis of neural circuits and mechanisms underlying the behavior shifting from positive to negative.

This study investigated the optogenetic stimulation of PVN–oxytocin neurons expressing channelrhodopsin-2 (ChR2) by selective ablation of MCH neurons in the LHA using a double transgenic mouse model expressing Cre recombinase (omb-cre) in both MCH and oxytocin neurons. We hypothesize that ablation of MCH neurons will decrease the expression of parental behavior in virgin male mice, even when PVN–oxytocin neurons are activated. The omb-cre mice are used to explore the relationship between LHA–MCH and PVN–oxytocin neurons associated with parenting and neglect behaviors.

## 2 Materials and methods

### 2.1 Animals

All experimental and animal housing protocols were approved by the Animal Care and Use Committee for Experimental Animal Ethics of Nippon Medical School with the guidelines for the Care and Use of Laboratory Animals of Nippon Medical School and National Institutes of Health Guidelines for the Care and Use of Experimental Animals. Sexually naïve male mice were housed in 19 × 27 × 15 cm polypropylene cages with clean wood chip bedding under controlled inversion illumination with a fixed 12/12 h light/dark cycle (lights on at 23:00), temperature (23°C), and humidity (50.0% ± 10%) with food and water provided *ad libitum*. All experiments were conducted during the dark phase. The inbred strain mice were bred in our laboratory.

### 2.2 Generation of MCH- and OXT-Cre double transgenic mice

Mice expressing Cre recombinase under the control of the MCH and oxytocin promoters, termed omb-cre mice, were established by breeding MCH-Cre mice [hemizygous for Tg (PMCH-Cre) 1 Lowl; JAX#014099] with OXT-Cre [Oxytocin-ires-Cre JAX#024234] mice. The adeno-associated virus (AAV) Helper-Free System (Agilent Technologies, Inc., Santa Clara, CA, United States) was used to produce and purify AAV vectors. The plasmids AAV9-EF1α-DIO-hChR2 (E123T/T159C)-EYFP were purchased from Addgene (ID: 44361, 44,362, 50,459, 35,509), while AAV9-CMV-FLEX-hrgreen florescent protein (hrGFP) was constructed starting with the AAV9-hrGFP plasmid (Agilent Technologies) and AAV9-CMV-FLEX-diphtheria toxin A fragment (DTA) from AAV9-MCS plasmid (Agilent Technologies). The efficacy of the latter constructs its cell-specific ablation was confirmed as previously reported (Inutsuka et al., 2016).

### 2.3 Stereotaxic AAV injection

To express ChR2 or GFP in PVN-oxytocin neurons, either the plasmid AAV9-EF1α-DIO-hChR2 (3 × 10^12^ copies/mL) or AAV9-CMV-FLEX-hrGFP (5.3 × 10^12^ copies/mL) were injected singly into the PVN (relative to bregma: anteroposterior, − 0.6 mm; mediolateral, ± 0.0 mm; dorsoventral, − 3.5 mm). For selective ablation of LHA-MCH neurons, plasmid AAV9-CMV-FLEX-DTA was bilaterally injected into the LHA (relative to bregma: anteroposterior, − 1.5 mm; mediolateral, ± 0.9 mm; dorsoventral, − 5.0 mm). All injections were performed using a stereotactic frame (Devid Kopf Instruments, Tujunga, CA, United States with Stoelting Mouse and Neonatal Rat Adaptor, Wood Dale, IL, USA), 27-gauge stainless steel tubing, and microprocessor-controlled syringe pumps (World Precision Instruments, Inc., Sarasota, FL, United States) at rate of 0.12 μL/min (600 nL/injection). All animals were injected with the indicated vector at 8 weeks of age and tested for parental behavior at 16 weeks.

### 2.4 Behavioral testing

All the experiments started 2 h after the dark phase and were videotaped (Sony HDR-cx670 HANDYCAM) in dim light. Before the test, cage containing a male mouse was moved from shelves to the observation area, where they habituated for 10 min to the experimental setting. Then the individual was exposed to a 5-min blue laser pulse (as described below) for pre-activating oxytocin neurons without presenting pups. The test started by placing three 4- to 7-day-old pups in a corner away from the male mouse, along with blue laser pulse optogenetics for 15 min. The distance recorded between the subject and the pups is defined as “close” as staying within proximity (< 1 cm) of pups. Parental behavior was assessed as previously reported (Orikasa et al., 2015). Parental behavior was assessed over 15 min by recording retrieving and crouching (defined as the limb extension and assuming a nursing-like posture over the pups). Burying behavior is defined as the mouse directing its head toward the pup and using its front limbs to scoop bedding toward the pup until it is completely covered by the bedding and no longer visible (Supplementary video). Sniffing latencies and time spent sniffing and close to pup were also recorded. Sniffing behavior is defined as sniffing or licking. All behaviors mentioned above were measured manually using event recorder software (gifted by Dr. Kondo). Partial ablation of LHA–MCH neurons was defined as an average remaining count of MCH neurons below 160.

### 2.5 Experimental design

**Figure 1:**
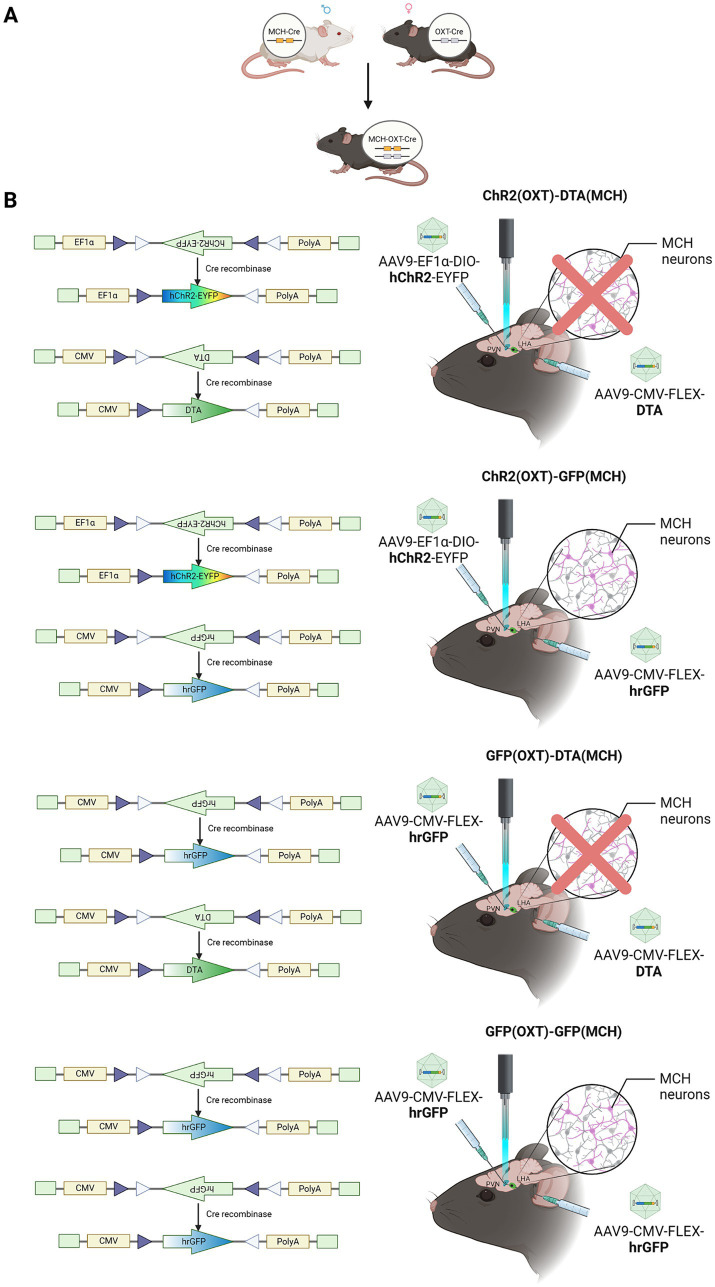
Experimental design schematic. **(A)** For establishing double transgenic mice, MCH-Cre and oxytocin-Cre mice were bred. **(B)** Virus schematic. Diagram representing the types of viral vectors used for different experimental conditions. ChR2 (OXT)-DTA (MCH), PVN–oxytocin neurons were injected with AAV9-EF1a-DIO-hChR2-EYFP to express ChR2 for optogenetic activation, LHA–MCH neurons were abolished by the injection with AAV9-CMV-FLEX-DTA; ChR2 (OXT)-GFP (MCH), PVN–oxytocin neurons were injected with AAV9-EF1a-DIO-hChR2-EYFP, LHA–MCH neurons were treated with AAV9-CMV-FLEX-hrGFP as control; GFP (OXT)-DTA (MCH), PVN–oxytocin neurons were injected with AAV9-CMV-FLEX-hrGFP as control, LHA–MCH neurons were injected with AAV9-CMV-FLEX-DTA for ablation; GFP (OXT)-GFP (MCH), both PVN–oxytocin neurons and LHA–MCH neurons were injected with AAV9-CMV-FLEX-hrGFP as control. [ChR2 (OXT)-DTA (MCH) group, *n* = 14 (12)], two individuals were excluded due to low ablation rate; [ChR2 (OXT)-GFP (MCH) group, *n* = 14]; [GFP (OXT)-DTA (MCH), *n* = 8 (5)], three individuals were excluded due to low ablation rate; [GFP (OXT)-GFP (MCH), *n* = 8].

The primary aim of this study was to examine the effect of MCH neurons ablation on the promotion of parental behavior by oxytocin neurons optogenetic activation. Sexually naïve male mice were divided into one of four experimental groups for this study. The first group was injected with ChR2 into the PVN following injection of DTA in the LHA [ChR2 (OXT)-DTA (MCH) group, *n* = 14 (12)], two individuals were excluded due to low ablation rate. The second group was injected with ChR2 into the PVN following injection of GFP in the LHA [ChR2 (OXT)-GFP (MCH) group, *n* = 14]. The third group was injected with GFP into the PVN following injection of DTA in the LHA [GFP (OXT)-DTA (MCH), *n* = 8 (5)], three individuals were excluded due to low ablation rate. The last group was injected with GFP into both the PVN and the LHA as a negative control [GFP (OXT)-GFP (MCH), *n* = 8] (Figures 1A,B).

### 2.6 Optogenetic procedures

All the surgical procedures were performed under anesthesia using a sodium 5-ethy1-5-(1-methylbutyl) barbiturate-xylazine cocktail. The animals were fitted with a single fiberoptic cannula (relative to bregma: AP, − 0.6 mm; ML, ± 0.0 mm; DV, − 3.5 mm) (diameter 200 μm, length 5 mm, COME2-FTR/C-F5, LUCIR Inc., Tsukuba, Japan) above the PVN for stimulation at least 1 week before behavioral testing. The optic fiber cable was then connected to the optical sleeve (ADAL1-5, Thorlabs, Inc.) to allow the animals to move around the test cage during testing. Optogenetic photostimulation was initiated 5 min before pup presentation by referring to our previous report (Kato et al., 2021) and continued throughout the 15-min behavior test. All groups were stimulated using the same optogenetic stimulation regimen. Briefly, blue laser pulse (473 nm, 10 ms, 30 Hz, 12 mW/mm^2^) was administered through the optic fiber during the test session.

### 2.7 Immunohistochemistry

Serial coronal sections (40 μm) were cut using a Microm HM 560 cryostat (Thermo Fisher Scientific, Waltham, MA, United States). All immunostaining steps were conducted at room temperature and on a plate shaker. Slices were first rinsed three times (10 min per wash) in 0.01 M PBS (pH 7.4) and incubated with polyclonal anti-MCH antibody in rabbit (1:5,000; H-070-47, Phoenix Pharmaceuticals Inc. Burlingame, CA, United Staes) or monoclonal anti-oxytocin antibody in mouse (1:200; gifted by Dr. Higashida) in blocking solution. The next day, slices were rinsed three times in PBS and incubated with Alexa Fluor 594-goat anti-rabbit/mouse IgG (both 1:200; molecular probe). After a final rinse in 0.01 M PBS, stained brain sections were then wet mounted on slides. Once dried, they were covered with mounting medium.

### 2.8 Statistical analysis

All data were analyzed using GraphPad Prism (GraphPad Software Inc.). Categorical variables were compared between groups by Chi-squared test followed by Fisher’s exact test for *post hoc* analysis. Continuous variables such as partial ablation of LHA–MCH neurons or the duration of each behavior were analyzed by one-way analysis of variance (ANOVA), with main factor LHA–MCH neurons’ ablation or two-way ANOVA with main factors PVN–oxytocin neurons’ activation and LHA–MCH neurons’ ablation, followed by Bonferroni’s *post hoc* tests for pairwise comparisons, respectively.

## 3 Results

**Figure 2:**
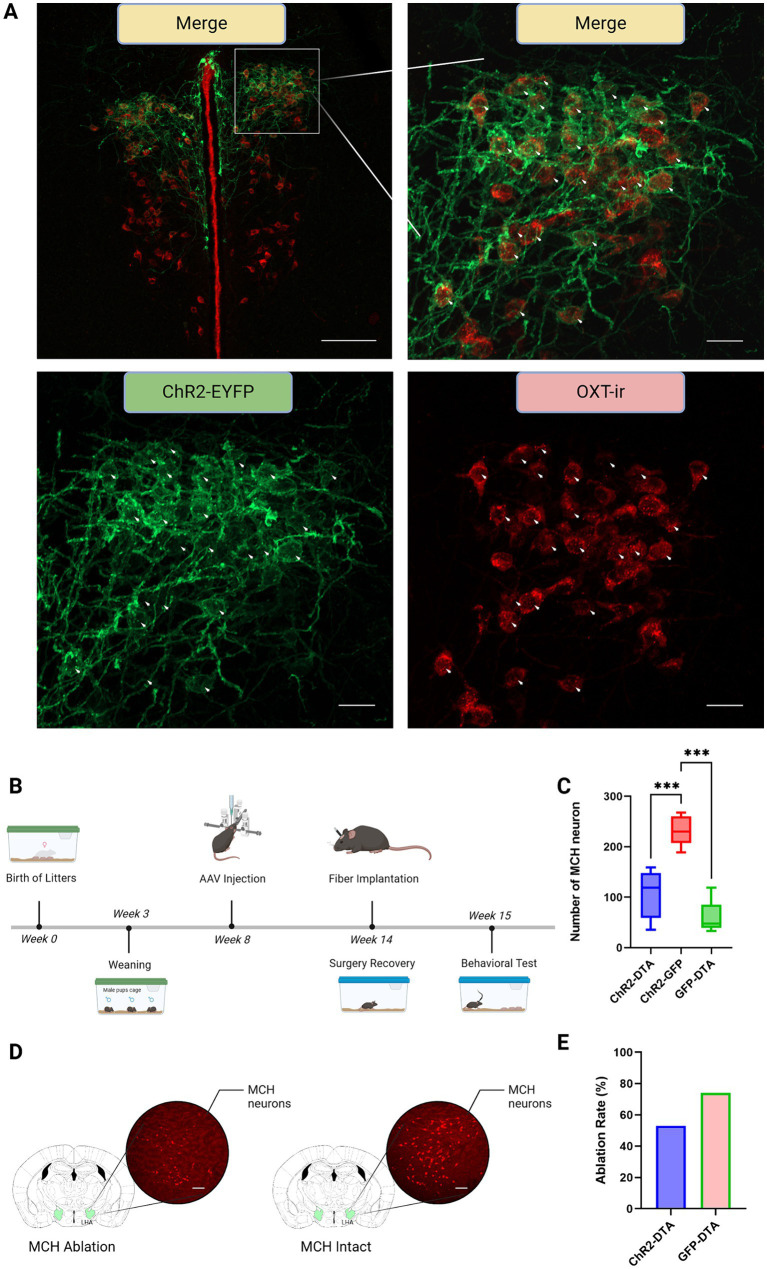
Immunohistochemistry and experimental schedule. **(A)** Upper panel left: merge at 10x magnification. The area enclosed by the white square represents the region magnified at 20x, as shown in the top right, bottom left, and bottom right. Green: paraventricular hypothalamic nucleus (PVN)–channelrhodopsin 2 (ChR2) expression neurons; Red: PVN–oxytocin immunoreactive (ir) neurons; Merge: ChR2 and oxytocin co-expression neurons. Scale bars, 100 μm. Upper panel right: merge at 20x magnification. Arrowheads: simultaneous visualization of PVN–ChR2 expression neurons and PVN–oxytocin-ir neurons. Scale bars, 20 μm. Lower panel left: PVN–ChR2 expression neurons. Scale bars, 20 μm. Lower panel right: PVN–oxytocin-ir neurons. Scale bars, 20 μm. **(B)** Graphic representation of total experiment schedule from birth to behavioral test commencement. **(C)** Graph showing the quantification of MCH-ir neurons after different treatments. Statistical analysis using one-way ANOVA followed by a Bonferroni *post hoc* test. **(D)** Left: LHA–MCH neurons injected with AAV9-CMV-FLEX-DTA for ablation; Right: LHA–MCH neurons injected with AAV9-CMV-FLEX-hrGFP as control. Scale bars, 100 μm. **(E)** The ablation ratio of LHA–MCH neurons in different groups compared to controls injected with AAV9-EF1a-DIO-hChR2-EYFP. *** *p* < 0.01.

A double transgenic sexually naïve male mouse model expressing Cre recombinase in both MCH and oxytocin neurons, which was generated by crossing MCH-Cre mice and oxytocin-Cre mice was used to demonstrate whether PVN–oxytocin neuron dependent parental behavior is contributed by LHA–MCH neurons (Figures 1A,B, 2A,B). Mice that received AAV9-CMV-FLEX-DTA in LHA–MCH neurons exhibited significantly low expression of LHA–MCH neurons compared with AAV9-CMV-FLEX-hrGFP injected (*F* (2,16) = 29.24, *p* < 0.001; Figures 2C,D). The ablation rate of LHA–MCH neurons in the ChR2-DTA and GFP-DTA groups was approximately 53 and 74%, respectively (Figure 2E).

**Figure 3:**
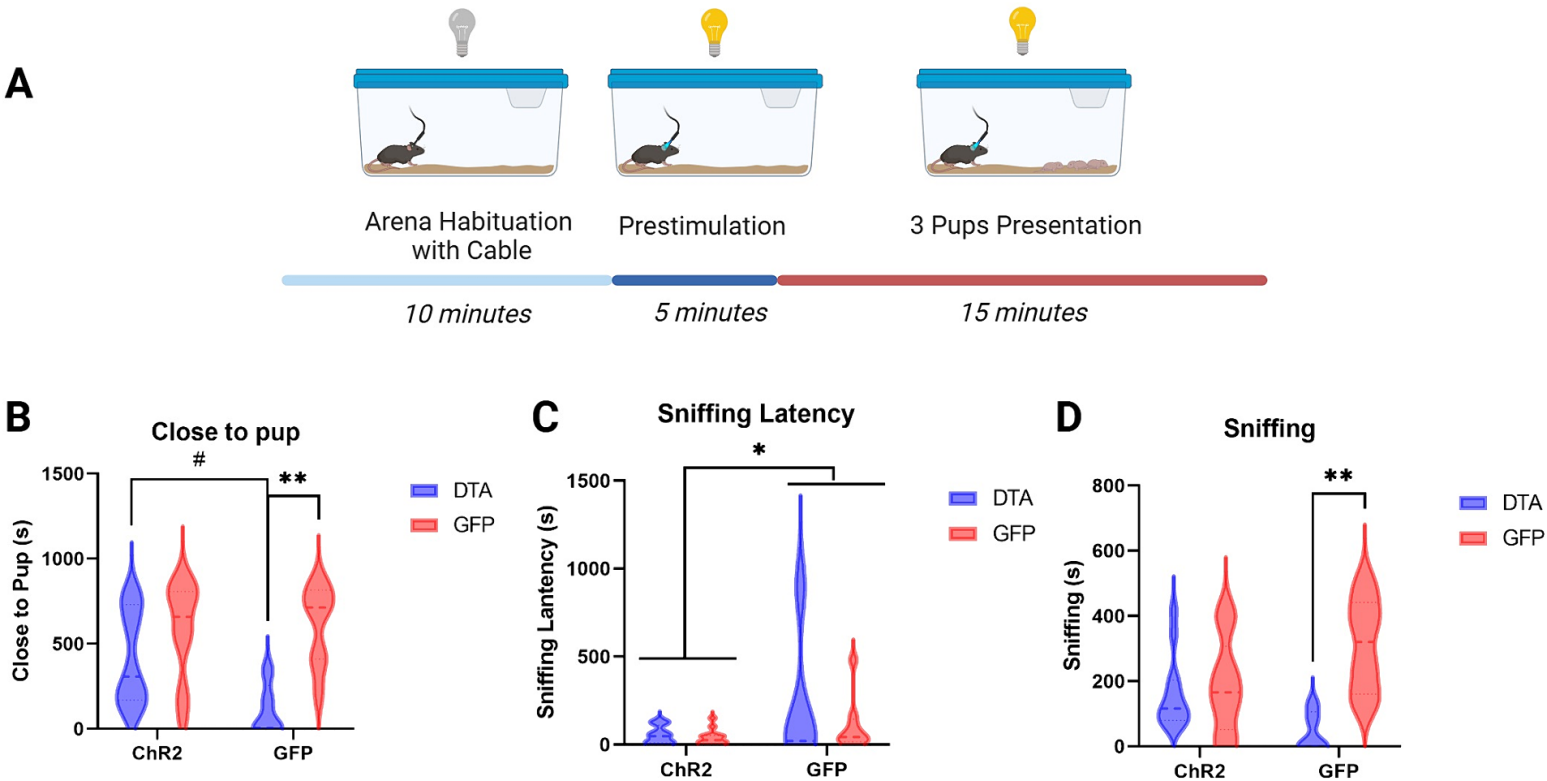
Behavior schematic and results. **(A)** Graphic representation of the behavioral test conducted after virus injection and fiber implantation, illustrating the setup and sequence of events. **(B)** Graphic showing the time spent close to pup (in seconds) after different treatments. Statistical analysis using two-way ANOVA followed by a Bonferroni *post hoc* test **(C)** Graphic depicting the latency until the onset of sniffing behavior following different treatments. Statistical analysis using two-way ANOVA followed by a Bonferroni *post hoc* test. **(D)** Graphic representation of the duration of sniffing (in seconds) after different treatments. Statistical analysis using two-way ANOVA followed by a Bonferroni *post hoc* test. [ChR2 (OXT)-DTA (MCH) group, *n* = 14 (12)], two individuals were excluded due to low ablation rate; [ChR2 (OXT)-GFP (MCH) group, *n* = 14]; [GFP (OXT)-DTA (MCH), *n* = 8 (5)], three individuals were excluded due to low ablation rate; [GFP (OXT)-GFP (MCH), *n* = 8]. # *p* < 0.1, * *p* < 0.05, ** *p* < 0.01.

In the behavioral test (Figure 3A), the mice with ablated LHA–MCH neurons, optogenetic stimulation of PVN–oxytocin neurons (ChR2-DTA) exhibit a tendency to increase the time spent close to pup compared with that in mice injected with GFP (GFP-DTA) (Figure 3B). Conversely, when PVN–oxytocin neurons were injected with GFP, which was not activated during the test, the ablation of LHA–MCH neurons (GFP-DTA) significantly decreased the time spent close to pup compared with that in the GFP-injected control group (GFP-GFP). A two-way analysis of variance (ANOVA), followed by a Bonferroni test for *post hoc* analysis revealed a significant main effect of LHA-MCH neurons’ ablation (*F* (1, 34) = 14.11, *p* < 0.001; Figure 3B).

Sniffing latency showed a similar pattern. Regardless of whether LHA–MCH neurons were ablated, activation of PVN–oxytocin neurons (ChR2-DTA, ChR2-GFP) significantly decreased the sniffing latency compared to groups with intact PVN–oxytocin neurons (GFP-DTA, GFP-GFP). A two-way ANOVA, followed by a Bonferroni test, revealed a significant main effect of PVN–oxytocin neurons’ activation (*F* (1, 34) = 4.62, *p* < 0.05; Figure 3C). The duration of sniffing showed comparable trends to time spent with pups. Ablation of LHA–MCH neurons along with intact PVN–oxytocin neurons (GFP-DTA) significantly decreased the duration of sniffing compared to the intact control group (GFP-GFP). No significant differences were observed among the other three groups (ChR2-DTA, ChR2-GFP, GFP-GFP). A two-way ANOVA followed by a Bonferroni *post hoc* test revealed a significant interaction between PVN–oxytocin neuron and LHA–MCH neuron treatments (F (1, 34) = 7.68, *p* < 0.01) and a significant main effect of LHA-MCH neurons’ ablation (F (1, 34) = 10.98, *p* < 0.01; Figure 3D).

**Figure 4:**
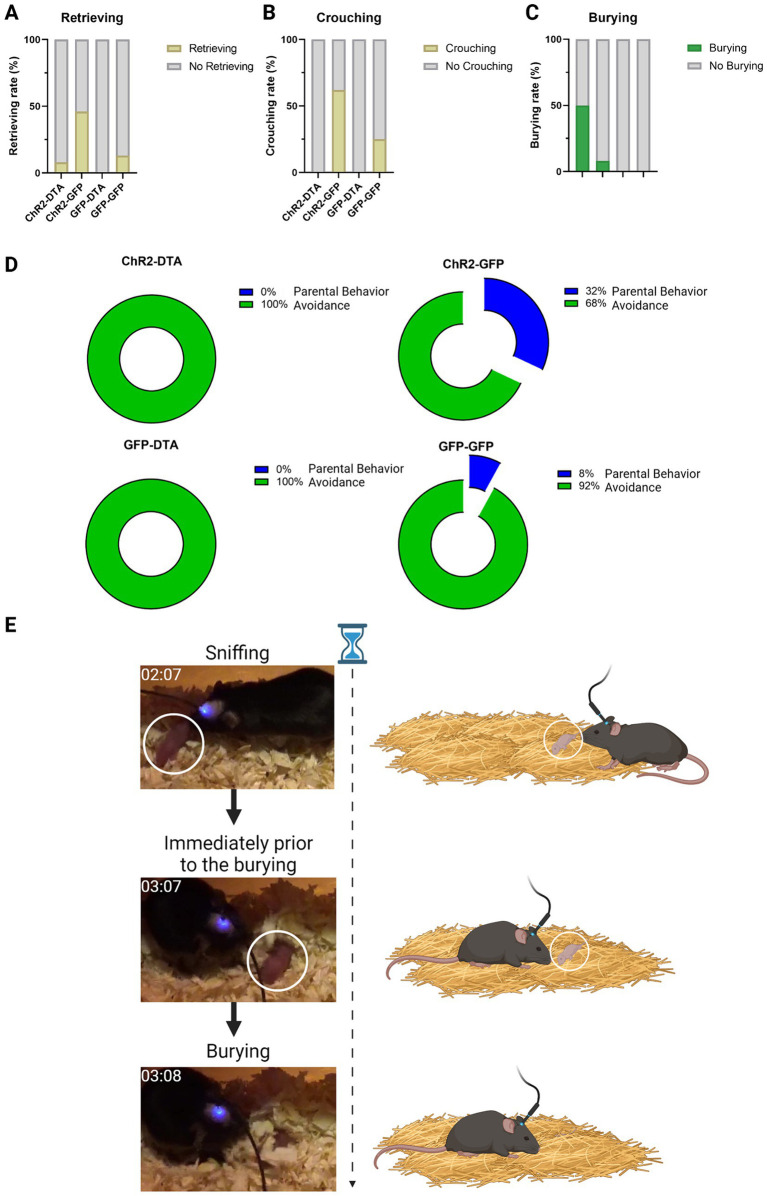
Parental behavior schematic and results. **(A)** Graphic displaying the rate of retrieving behavior after different treatments. Statistical analysis using a Chi-square test followed by Fisher’s exact *post hoc* test. **(B)** Graphic showing the rate of crouching following different treatments. Statistical analysis using a Chi-square test followed by Fisher’s exact *post hoc* test. **(C)** Graphic showing the rate of burying after different treatments. Statistical analysis using a Chi-square test followed by Fisher’s exact *post hoc* test. **(D)** Graphic representation of the percentage composition of parental behavior and avoidance following different treatments. **(E)** Graphic representation of the instance when subjects engaged in burying behavior toward the pups.

Furthermore, activation of PVN–oxytocin neurons with intact LHA–MCH neurons (ChR2-GFP) significantly increased crouching behavior compared to the group with only LHA–MCH neurons ablated (GFP-DTA). Mice with activated PVN–oxytocin neurons and ablated LHA–MCH neurons (ChR2-DTA) exhibited significantly decreased crouching behavior compared to the LHA–MCH neurons intact group (ChR2-GFP). These findings suggest that without MCH neuronal regulation, even the activation of PVN–oxytocin neurons will not suffice to induce typical parental behavior. There were no significant differences regarding retrieving behavior. These results were determined by a Chi-square test followed by Fisher’s exact *post hoc* test (χ^2^ = 14.40, df = 3, *p* < 0.01; Figures 4A,B).

Intriguingly, mice with ablated LHA–MCH neurons but activated PVN–oxytocin neurons (ChR2-DTA) exhibited significantly increased burying behavior toward pups compared with mice in which both PVN–oxytocin and LHA–MCH neurons were intact (GFP-GFP) or only PVN–oxytocin neurons were activated by ChR2 with intact LHA–MCH neurons (ChR2-GFP). There was no significant difference in this behavior between the ChR2-DTA and GFP-DTA groups. These results were confirmed by a Chi-square test followed by Fisher’s exact *post hoc* test (χ^2^ = 11.89, df = 3, *p* < 0.01; Figure 4C). We defined the average time spent on burying and moving away from the pups as “Avoidance” and the time spent on retrieving and crouching as “Parental behavior” and then calculated the ratio of these behaviors (Figures 4D,E).

## 4 Discussion

In this study, a double transgenic mouse line, omb-cre mouse, was established to enable stimulation of oxytocin neurons in the presence and absence of MCH neurons input. Parental behavior promoted by the activation of oxytocin neurons differed markedly in the presence and absence of MCH neurons input. Specifically, the partial ablation of MCH neurons reduced protective parental behavior and increased defensive burying behavior, suggesting that the increased burying behavior towards pups results from insufficient MCH neurons input to oxytocin neurons in the PVN.

Photostimulation of the ChR2-expressing PVN–oxytocin neurons with intact LHA–MCH neurons could increase parental behavior (Marlin et al., 2015). However, the same photostimulation regimen did not promote parental behavior in male mice with ablation of MCH neurons. Kato et al. (2021) Reported that complete ablation of MCH neurons reduced parental crouching behavior. However, complete ablation increased lethal aggression against pups by virgin males, whereas in the present study, there was no infanticide event at almost 50% ablation of MCH neurons. Therefore, the magnitude of the shift from protective to aggressive parental behavior may depend on the extent of MCH neurons loss.

The MCH system comprises MCH receptor 1 (MCHR1) and MCH receptor 2 (MCHR2), both of which are G-protein-coupled receptors, with only MCHR1 being widely expressed in rodents (Presse et al., 2014). This system plays a critical role in regulating feeding behavior and energy balance (Karlsson et al., 2012; Presse et al., 2014) and is also implicated in social motivation, including parental behavior. A previous study reported that MCH administration increased social interactions directed toward juveniles (Barretto-de-Souza et al., 2023). In the current study, partial ablation of the LHA–MCH neurons decreased the duration of close to pup and sniffing time, suggesting that LHA–MCH neurons are important for social interaction. Both postpartum and virgin female mice with knockout of MCHR1 (MCHR1 KO) also exhibited poor parental behavior (Alachkar et al., 2016; Alhassen et al., 2019), suggesting that intact PVN–oxytocin neurons can no longer promote beneficial parental behavior in the absence of sufficient neuromodulation by LHA–MCH neurons.

Oxytocin is a modulator of social behavior in both humans and animals (Donaldson and Young, 2008) and a critical regulator of mating, parturition, and lactation in females, otherwise pair-bonding, maternal–infant bonding, and maternal attachments, which in turn are implicated in social rewards and reinforcement. Sensory information, particularly auditory information from the pups, plays a crucial role in triggering parental behavior. A previous study showed that oxytocin neurons responded to pup vocalizations through input from the posterior intralaminar thalamus, and repetitive thalamic stimulation induced lasting disinhibition of oxytocin neurons (Valtcheva et al., 2023). The present study also demonstrated that the initiation of parental behavior requires the normal functioning of both LHA–MCH and PVN–oxytocin neurons.

Because MCH typically exhibits inhibitory actions, it can reduce presynaptic neurotransmitter release (Gao and van den Pol, 2001) and decrease postsynaptic membrane potential, thereby attenuating spike frequency (Wu et al., 2009). MCH neurons contain GABA (Beekly et al., 2023) and are also connected to oxytocin neurons (Kato et al., 2021). Administration of oxytocin intraperitoneally or optogenetically activates PVN–oxytocin neurons can efficiently induce parental behavior (Marlin et al., 2015). It was also shown that appropriate GABA disinhibition in PVN–oxytocin neurons triggers the release of oxytocin during lactation (de Kock et al., 2003). These studies suggest that MCH neurons contribute to the fine modulation of oxytocin neurons associated with parental behavior.

It has been shown that oxytocin can selectively excite LHA–MCH neurons through mechanisms involving the Na^+^/Ca^2+^ exchanger and nonselective cation channels (Yao et al., 2012). Based on these interactions, it can be speculated that in mice with intact LHA–MCH neurons, the activation of PVN–oxytocin neurons subsequently excite LHA–MCH neurons. Therefore, LHA–MCH neurons can act downstream of PVN–oxytocin neurons because the ablation of MCH neurons prevents parental behavior by the photostimulation of oxytocin neurons. Furthermore, the optogenetic stimulation of ChR2-expressing fibers from MCH neurons also promotes parental behavior (Kato et al., 2021). Sires must preserve similar neural circuits and exhibit similar behaviors as observed in virgin males.

Additionally, partial ablation of MCH neurons also shifted the response of PVN photostimulation from protective/beneficial to defensive burying behavior of pups. Burying behavior occasionally occurs in response to stress as observed in marble-burying or shock-probe burying tests, which are screening tests for anxiolytic response or autism spectrum disorder (Angoa-Pérez et al., 2013; Nicolas et al., 2006). The ubiquity of this behavior suggests substantial survival value. In a previous study, central infusion of oxytocin alone reduced marble-burying behavior, whereas antagonizing the MCH system blocked this oxytocin-mediated reduction (Sanathara et al., 2018). Although our results indicate that LHA–MCH neurons are critical for regulating PVN–oxytocin neuron dependent parental behavior, disrupting this pathway can induce negative pup-directed burying behavior. In the current study, we have employed an AAV virus to specifically ablate LHA-MCH neurons. However, varied infection rates could influence the consistency of neuron ablation across subjects. Future studies might benefit from alternative methods, such as the Tet-off system, to enhance the precision of neuron ablation.

Activation of oxytocin neurons in the mouse PVN can promote parental behavior, whereas partial ablation of LHA–MCH neurons projecting to the PVN induces a shift from protective to negative paternal behavior. Therefore, the interaction between LHA–MCH and PVN–oxytocin neurons is crucial for initiating appropriate parental behavior.

## Data Availability

The original contributions presented in the study are included in the article/Supplementary material, further inquiries can be directed to the corresponding author.
